# New insights and innovation from a million crystal structures in the Cambridge Structural Database

**DOI:** 10.1063/1.5116878

**Published:** 2019-08-28

**Authors:** Jason C. Cole, Seth Wiggin, Francesca Stanzione

**Affiliations:** The Cambridge Crystallographic Data Centre, 12 Union Road, Cambridge CB2 1EZ, United Kingdom

## Abstract

The Cambridge Structural Database (CSD) is the world's largest and most comprehensive collection of organic, organometallic, and metal-organic crystal structure information. Analyses using the data have wide impact across the chemical sciences in allowing understanding of structural preferences. In this short review, we illustrate the more common methods by which CSD data influence molecular design. We show how more data could lead to more refined insights into the future using a simple example of trifluoromethylphenyl fragments, highlighting how with sufficient data one can build a reasonable model of geometric change in a chemical fragment with torsional rotation, and show some recent examples where the CSD has been used in conjunction with other methods to provide design ideas and more computationally tractable workflows for derivation of useful insights into structural design.

## INTRODUCTION

The Cambridge Structural Database (CSD)^1^ is a large collection of crystal structures; a recent milestone passed in June 2019 was the release of the one millionth structure to the community,^2^ an N-heterocycle synthesized by chalcogen-chalcogen bonding catalysis.^3^ The large resource of structures has had an impact since the Cambridge Crystallographic Data Centre's (CCDC) inception in 1965, but the wealth of information now available has the potential to be more transformative in the coming years. Of the top 200 pharmaceutical products in 2018, 124 are small molecule compounds.^4,5^ Of these, 70 have an exact match to a crystal structure in the CSD. Small molecule structures are generally more precise and more accurate than protein structures due to their higher resolution, which allows users to gain detailed insights into molecular geometry and molecular interactions.

In this short review, we show some examples where the CSD is used in drug discovery, demonstrate a simple example where the additional information now available allows additional insight, discuss new methods to interrogate the CSD, and highlight some examples where access to this large resource is allowing innovation through screening and machine learning in other fields. In particular, we show an example where sufficient data mean we can now understand how PhCF_3_ fragment's valence angles are likely to change as a function of torsional rotation; a process we might expect to see in the dynamic motion of such fragments.

## HOW THE CSD IS APPLIED IN DRUG DISCOVERY

**FIG. 1.**
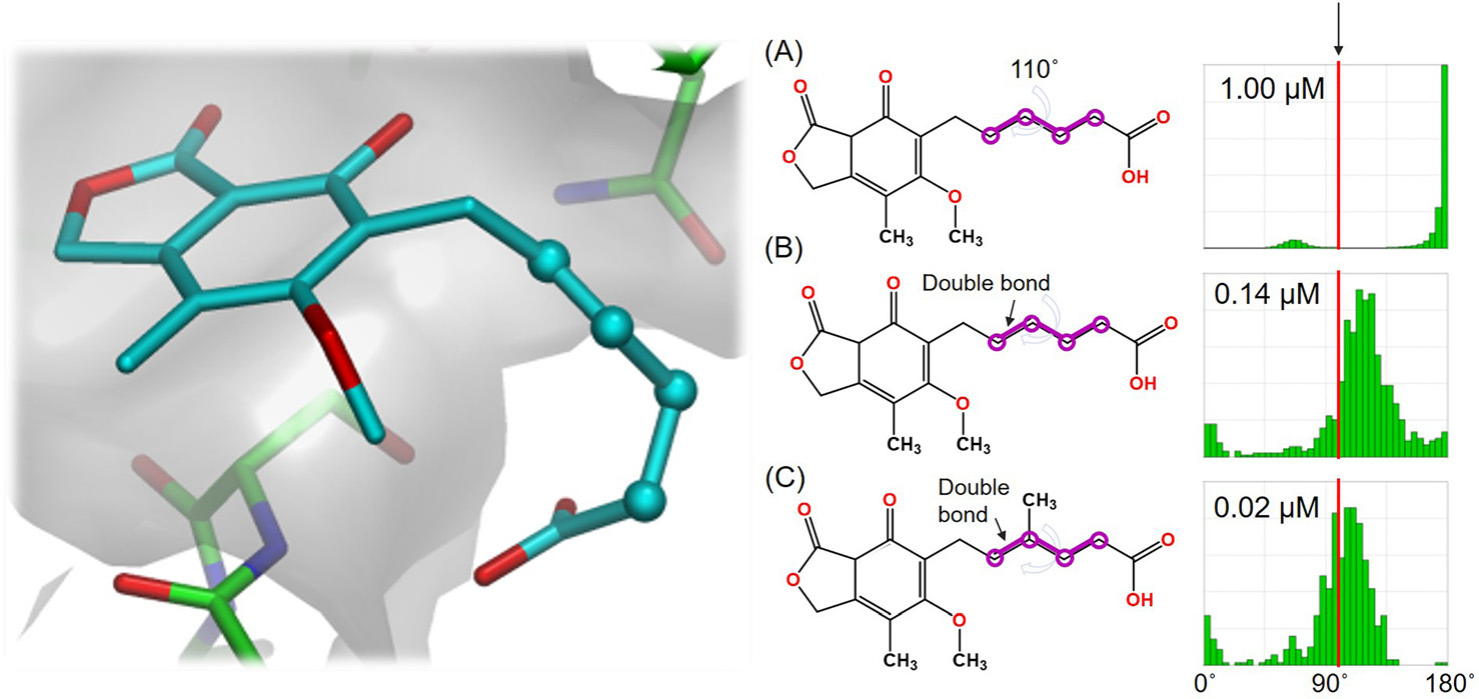
Inosine monophosphate dehydrogenase (IMPDH) binder optimization. The original ligand (a) binds with a torsion angle of 110°. On the right, the observed distributions of similar torsion angles in the CSD are shown (taken from Mogul). By chemical change (going from A to B to C), one can see that the observed torsion angle (in red) is better aligned with CSD observations. The consequence is reduced strain in the inhibitor and increased bioactivity (shown as IC_50_ values of binding to inosine IMPDH).

The most common use of the CSD in drug discovery is in the analysis of conformation. Brameld and co-workers have published an excellent and comprehensive review on using conformational information in drug discovery highlighting how CSD information can be very useful in making design decisions.^6^ In one example highlighted in this paper, the authors show how understanding conformational preferences can be used to optimize the binding of an inosine monophosphate dehydrogenase (IMPDH) inhibitor by understanding geometric strain (see Fig. 1). Mogul^7^ allows easy analysis of the torsional preferences and can be used to help make inform decisions on molecular design to reduce internal molecular strain. Such examples are frequent in the medicinal chemistry literature. For example, the CSD has been used to analyze substituent effects in benzamides,^8^ in the design of selective benzoxazepin PI3Kδ inhibitors,^9^ and in the identification of a selective, nonprostanoid EP2 receptor agonist.^10^

The CSD can also be used to understand intermolecular interactions. The IsoStar^11^ database contains information about a wealth of interactions in both the CSD and the Protein Data Bank^12^ (PDB). Using the information in IsoStar can allow users to rationalize changes in affinity due to contacts within protein ligand systems. In one example, Certal and co-workers^13^ rationalized an increase in affinity on binding due to N···S contacts in the observed protein ligand complex. They found that the contact was more frequent than one would expect by chance based on observations from the CSD. Intramolecular interactions too can be studied; for example, Kuhn and co-workers published a seminal review of intramolecular hydrogen bonding for medicinal chemists based (in part) on observations taken from the CSD.^14^

Finally, information in the CSD can be used as a data source for knowledge-based predictive algorithms. For example, the CSD has been used in various approaches for the generation of conformational ensembles, which are of general utility.^15,16^ Similarly, scoring functions for molecular docking have been derived by the analysis of interactions in the CSD.^17^

## THE BENEFIT OF MORE INFORMATION: A SIMPLE CASE STUDY OF THE CONFORMATION OF TRIFLUOROMETHYL GROUPS BOUND TO PHENYL RINGS

Historically, users of the CSD have used informatics to interpret and understand conformational behavior. More data allow analyses that can reveal more detail. By way of example, we can take a simple case to illustrate how more data allow higher confidence and deeper insight into structural trends in crystalline systems.

**FIG. 2.**
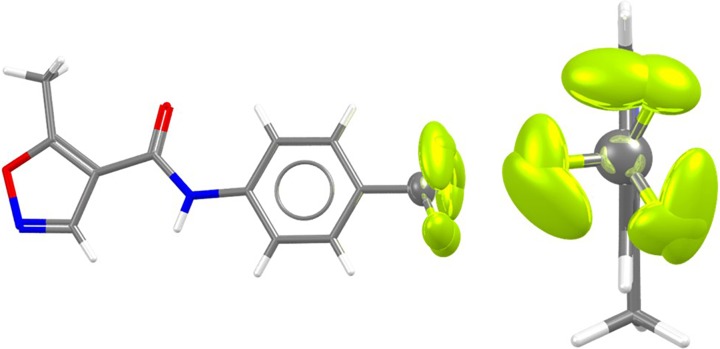
Rotational disorder around the CF_3_ group of polymorph II in Leflunomide (CSD refcode VIFQIL01). The CF_3_ group has been refined using two alternate conformations in the lattice. The anisotropic displacement parameters, in turn, suggest additional motion around the Ph-CF_3_ rotatable bond within the respective potential wells.

Trifluoromethylphenyl groups occur more frequently in small molecule crystal structures than they have historically (the CSD v5.40 May 2019 contains 2043 structures with such a group; only 298 of these were in publications predating 2006 in the 382 652 structures that were then available. The remaining 1745 have occurred since then in the subsequent 626 489 structures). Crystallographers are well aware that such groups are often disordered within a lattice, and indeed often occupy multiple conformations within the solid state [see, for example, the structures of both polymorphs of Leflunomide (CSD refcode family VIFQIL,^18^ shown in Fig. 2) both show rotational disorder around CF_3_ groups within the lattice]. Higher quality structures (organic, not disordered, single crystal structures with an R-factor <5%) are far rarer: only 276 structures from the 2043 structures with only 13 structures occurring before 2005 in this set.

Now we have significantly more data in the CSD, we can undertake more detailed analysis of the nature of such fragments based on higher quality structures. In the case of trifluoromethylphenyl, we can use the wealth of information to understand not only conformational preferences in the solid state, but also how the conformation of a CF_3_ group is related to the preferred values of the valence angles within the fragment.

**FIG. 3.**
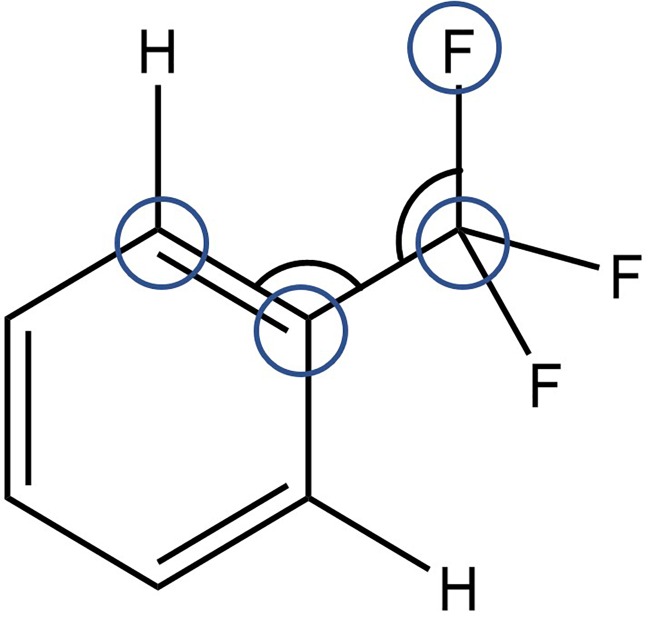
Search query parameters in the CSD: F-C(sp3)-C(ar)-C(ar) torsion angle, and F-C(sp3)-C(ar) and C(ar)-C(ar)-C(sp3) bond angles.

**FIG. 4.**
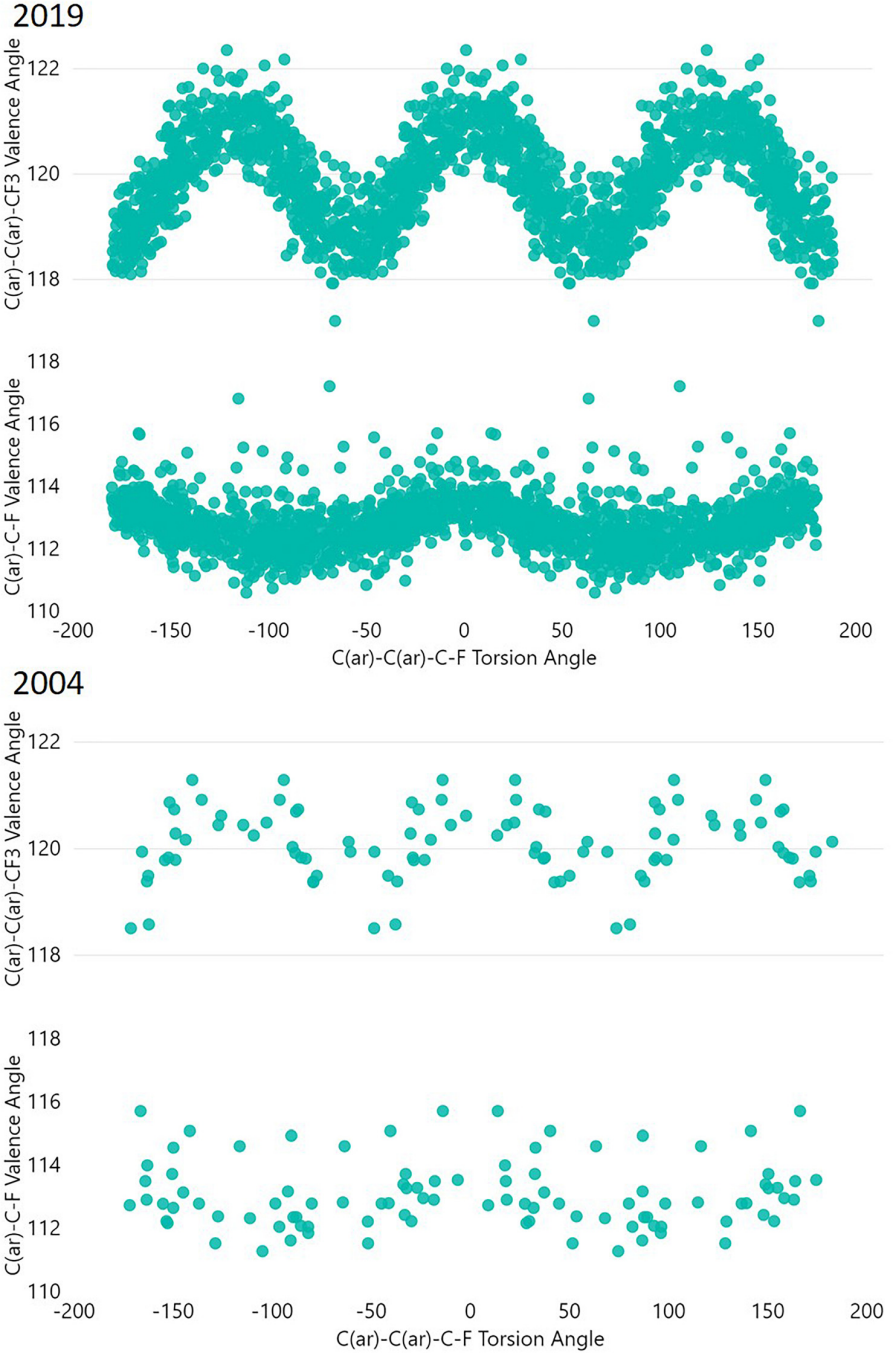
Variation of internal valence angles in PhCF_3_ groups with torsional rotation. At the top, results for all high precision structures are shown (organic, R-factor < 5%; no errors), at the bottom results are shown for structures up to 2004. All permutationally equivalent observations generatable from each detected fragment are included.

In Fig. 3, a query is shown that uses all the data in the current version of the CSD to characterize the motions of the CF_3_ group with respect to the conformation around the Ph-CF_3_ bond. We can analyze multiple parameters within the fragment (see Fig. 4). What becomes apparent from a CSD analysis is, first, CF_3_ groups in PhCF_3_ fragments are quite conformationally free: the torsion profile shows only a slight tendency toward any given torsion angle.

Some CF_3_ groups have a fluorine atom in the plane of the phenyl ring. These fluorine atoms are relatively close to a proximal hydrogen atom on the phenyl ring causing angular distortion in the plane of the aromatic ring. The C(ar)-C(ar)-C valence angle flexes most significantly, but in addition we see an additional distortion of the C(ar)-C-F angle. Both are larger to relieve the F···H clash.

Figure 4 also highlights the observed distributions for structures published before 2005. While the plot undoubtedly has the same trends, the sparsity of data would have led to a less certain conclusion being drawn in 2004; in 2004, we could have concluded that the CF_3_ torsion angle shows no strong conformational preference in the solid state. We can see suggestions toward how the internal angles vary with CF_3_ rotation too in the 2004 plot, but in 2019 we can understand how the conformation occupied influences the internal angles within the PhCF_3_ system in far more detail.

## NEW WAYS OF SEARCHING THE CSD

### CSD-CrossMiner

A wealth of data requires powerful methods for searching. CCDC has provided software systems to search and analyze information in the CSD.^19,20^ Most recently, effort has been made to provide newer methods for searching, including more elaborate pattern searching using a pharmacophore-like-representation of information within both the Protein Data Bank (PDB)^12,21^ and the CSD.^22^ CSD-CrossMiner is a powerful method for interactively searching based on predefined features. The method allows for searching of 3D geometric arrangements of features based SMARTS-pattern^23^ feature definitions.

**FIG. 5.**
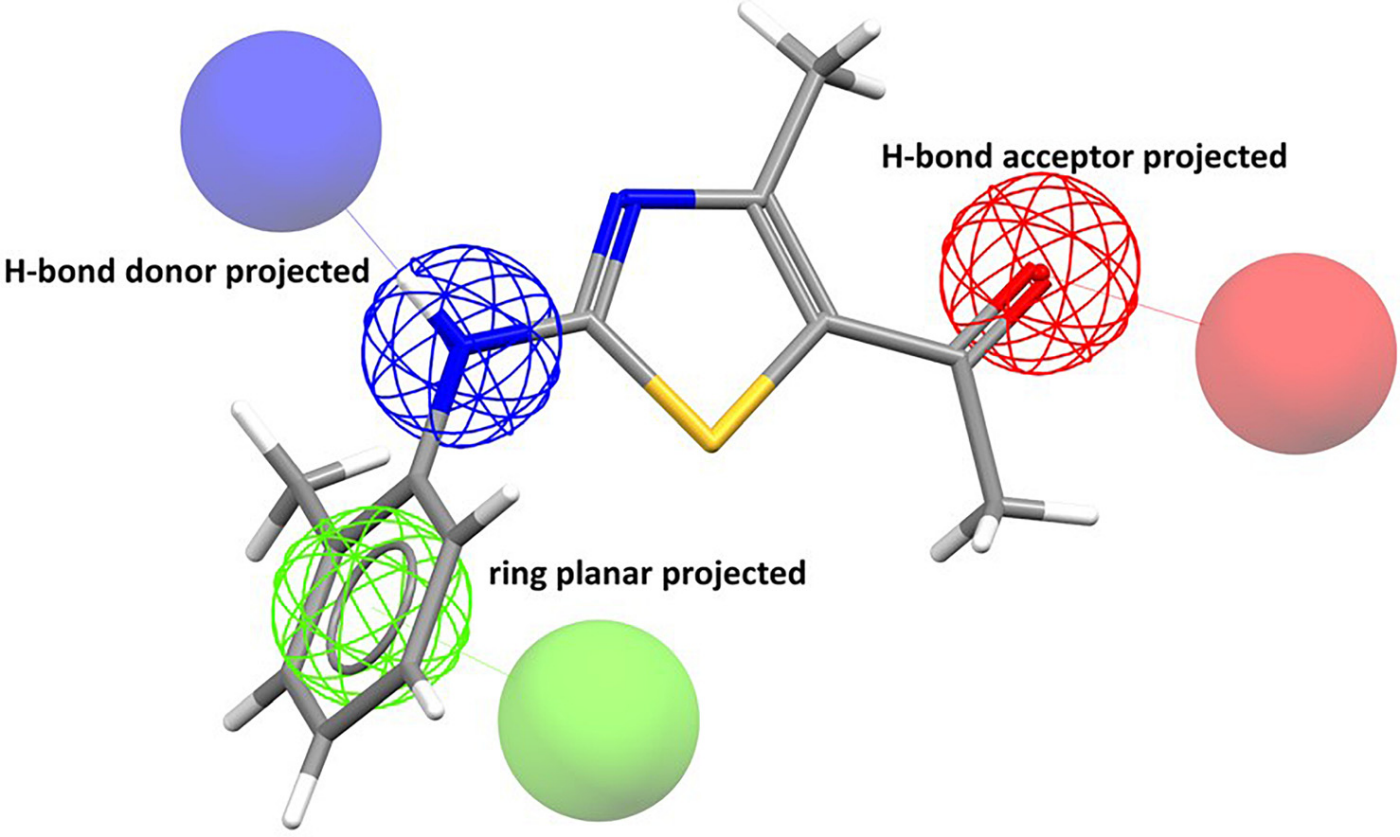
A typical pharmacophore search query in CSD-CrossMiner.

Figure 5 shows an example taken from a recent showcasing white paper,^24^ showing a fairly typical query. CSD-CrossMiner allows searching of the CSD based on a more abstract representation of chemistry that is more representative of traditional medicinal chemistry thinking of pharmacophores. In addition to the built-in features, the user can define their own more features using SMARTS patterns.

Searches using CSD-CrossMiner can be used to identify structurally similar pockets in proteins. The white paper shows in addition how the software can be used for understanding cross-reactivity, finding new scaffolds based on 3D information in the CSD and to find possible bioisosteric replacements. The software has been used in pharmaceutical compound design projects;^25^ the value of the ability to query interaction patterns for informing fragment based discovery has also been noted.^26^

### Programmatic access via a Python application programming interface

Another recent addition to the suite of methods for searching the CSD has been a Python based application programming interface (API).^27^ The API allows versatile searching of the CSD as end users can create customized scripts. The ability to access the data via scripts in tandem with other packages such as RDKit^28^ is very convenient for more advanced analysis of structural data as indicated by several recent examples.^16,29–34^

Researchers have been able to develop a very useful subset of the CSD where the molecules were deemed druglike.^35^

**FIG. 6.**
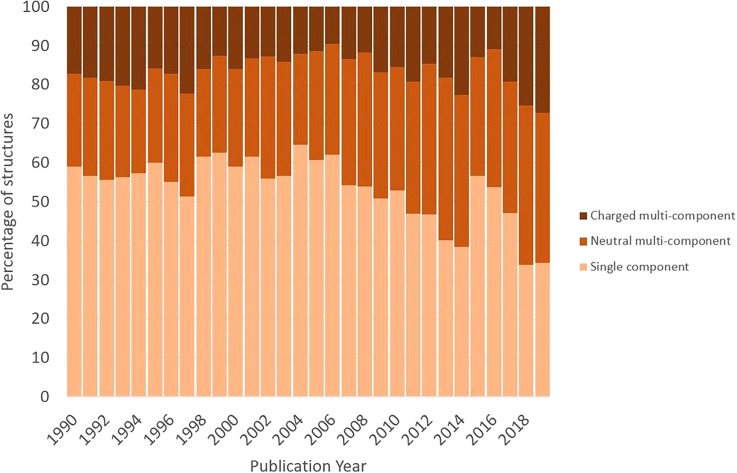
How multicomponent crystalline formulation has changed with time.

This drug subset in turn facilitates further analysis and comparison with the full CSD.^36^ For example, the molecules in the subset typically have a lower formula weight than all organic molecules from the CSD. Comparison of the number of hydrogen bond donors and acceptors for an entry in the subset compared to an “average” organic CSD entry is also informative; it shows that a smaller proportion of the druglike molecules in the CSD have no hydrogen bond donors or acceptors. It also shows that fewer druglike molecules are observed with large numbers of donors or acceptors, broadly agreeing with Lipinski's rules^37^ in this area. Differences in the elemental composition can also be seen, with druglike molecules less likely to contain phosphorus and also favoring lighter halogens (F and Cl over Br and I). It is also interesting to observe changing trends within the druglike molecules deposited in the CSD over time. It can be seen over the past thirty years that an increasing number of structures are multicomponent (either cocrystals or salts), with the percentage of single component structures dropping from around 55% in the early 1990s to less than 40% today (see Fig. 6).

Many research projects are benefitting from API access: For example, users have been able to more easily use machine learning in tandem with the API for solvate prediction,^38^ to help implement fragment pocket analysis using structural informatics,^39^ and to aid with crystal structure prediction,^29^ for understanding of the impact of compression of cocrystals^40^ (of interest in the formation of tablets) and for parametrization of structural refinement programs.^41^

### Looking to the future: Opportunities for the community

The CSD contains over a million structures and continues to grow. The plethora of data means users have new opportunities available to them. We have noted that this, combined with the programmatic access to the data now available, and cheap computational power is leading to studies that would not have been tractable in the past. For example, a recent study showed how end users could effectively apply a virtual screen of the CSD to find potential high carrier mobility organic semiconductors.^42^ In this study, the authors combined data mining with various levels of quantum theory calculations to mine the CSD and find promising “pre-existing” compounds, developed for use in other areas of chemistry, that may in fact act as good candidates in this space.

The CSD can be regarded as a “big data” resource, and as such there is renewed interest in making use of the information in the CSD to solve complex problems. One interesting example of machine learning in tandem with CSD data and quantum mechanical calculations was undertaken to try to create a rapid prediction mechanism for solid state Nuclear Magnetic Resonance (NMR) shifts.^43^ In this research, the authors used a set of 2000 diverse structures in the CSD along with solid state Quantum Mechanics (QM) based calculations (using GIPAW^44^) to create a training set to train a gaussian progress regression model for prediction of solid state NMR shifts. The model performs acceptably with test systems but is between 4 and 5 orders of magnitude quicker than using full QM calculations.

We should expect more machine-learned models of this type that will facilitate more rapid analysis of the solid state.

One opportunity and challenge for the community of users will be the need for more meta-data associated with structures, as such additional data will facilitate more data driven predictive modeling. Some authors are already approaching this challenge with text mining for annotation of metal-organic framework structures.^30^

The CCDC is working toward increasing the volume of meta-data associated with structures. Two notable recent changes are the inclusion of atomic displacement parameters, which aid structural interpretation, and the inclusion of the structure factors when provided by depositors. Such information has the potential for aiding validation, but in addition may be useful for prospective analysis. In addition, depositors can now link to raw crystal structure data by including a data document object identifier during deposition. Rhetorically, we can wonder what hidden insights may be available to a researcher prepared to return to the raw crystal structure data in the future?

## CONCLUSIONS

The CSD has grown to a remarkable one million structures since its inception in 1965. These structures have had a profound impact across the community, with significant impact in drug discovery and drug development. The chemical coverage of compounds in the CSD increases year-on-year as new classes of compounds are synthesized and crystallized. As the volume of data has increased more detailed insights from data have become discernible. We look forward to the next million structures and the insights they will provide.

## SUPPLEMENTARY MATERIAL

See the supplementary material for underlying individual search results generated by the ConQuest search to generate the data points in Fig. 3.
